# Anti-Inflammatory Effect of Vanillin Protects the Stomach against Ulcer Formation

**DOI:** 10.3390/pharmaceutics14040755

**Published:** 2022-03-30

**Authors:** Murilo Piologo Ciciliato, Matheus Chiaradia de Souza, Carolina Mendes Tarran, Ana Laura Tironi de Castilho, Ana Júlia Vieira, Ariane Leite Rozza

**Affiliations:** Department of Structural and Functional Biology, Institute of Biosciences, São Paulo State University (UNESP), Dr Antonio Celso W Zanin Street 250, Botucatu 18618-689, Brazil; Murilo.ciciliato@unesp.br (M.P.C.); Matheus.chiaradia@unesp.br (M.C.d.S.); Mendes.tarran@unesp.br (C.M.T.); Ana.tironi@unesp.br (A.L.T.d.C.); Julia.vieira@unesp.br (A.J.V.)

**Keywords:** vanillin, stomach, inflammation, ELISA, qPCR, TNF-α, IL-1β, IFN-γ, NF-κB, NO

## Abstract

Gastric ulcer is one of the most frequent gastrointestinal disorders, and there is an increasing search for natural products that can heal ulcers and avoid their recurrence. We aimed to evaluate the gastroprotective activity of vanillin, including the investigation of anti-inflammatory activity and the modulation of gene expression. Wistar rats were orally treated with vehicle, carbenoxolone, or vanillin (25, 50, or 100 mg/kg) and orally received absolute ethanol to develop gastric ulcers. We analyzed the ulcer area, conducted histological analysis, and measured the levels of the inflammatory cytokines TNF-α, IL-6, IL-1β, and IFN-γ, and anti-inflammatory cytokine IL-10 by ELISA. We analyzed mRNA expression for NF-κB, TNF-α, and Il-10. We measured NOx levels using the Griess reaction. Our results showed similar gastroprotection for the three doses. Vanillin increased mucus production and preserved gastric mucosa integrity. The gastroprotective effect was linked to anti-inflammatory activity as a result of decreasing the levels of TNF-α, IL-6, IL-1β, and IFN-γ and increasing IL-10 levels. Vanillin downregulated the mRNA expression of NF-κB and TNF-α, upregulated the mRNA expression of Il-10, and increased NOx levels in the stomach. The gastroprotective activity of vanillin is related to the maintenance of gastric mucus and the local inflammatory response modulation.

## 1. Introduction

Gastric ulcer is an inflammatory disease with a high economic impact [1]. It is estimated that about 15 million people worldwide are affected, with a mortality rate higher than 4 million people per year [2]. This disease is associated with an imbalance between gastroprotective factors [3] and harmful agents [4]. The gastroprotective factors include mucous secretion, decreased gastric juice production, adequate blood flow in the submucosa, and the production of anti-inflammatory and antioxidant proteins, which increases the ability of the mucosa to preserve itself even in contact with ulcerogenic agents. In addition to a genetic predisposition, nonsteroidal anti-inflammatory drug consumption, alcohol consumption, *Helicobacter pylori* infection, and stress are the primary agents of the gastric ulcer [5].

Alcohol consumption is a leading cause of gastric ulcer development [6]. In contact with the gastric mucosa, ethanol promotes neutrophil infiltration in the submucosal layer of the stomach, stimulating the formation of free radicals and triggering an inflammatory cascade. These events give rise to an oxidative stress environment [7], which causes vascular damage and induces gastric cell necrosis [3].

In preclinical studies, absolute ethanol is widely used to induce gastric ulcers, using rats or mice as the experimental model. Ethanol intake directly damages the gastric mucosa and triggers excessive production of ROS, which leads to an inflammatory response in the gastric mucosa [8]. NF-κB, a redox-sensitive transcription factor, mainly regulates inflammatory mediators. Once activated, this cytoplasmic complex translocates to the nucleus and stimulates the transcription of inflammatory mediators, such as the cytokines TNF-α, IL-6, and IL-1β [9,10]. Therefore, regulating factors involved in the inflammatory response is essential for gastric ulcer prevention and treatment.

Some commercial drugs are widely used to treat gastric disturbances, but their use is associated with several side effects [11], including the risk of developing gastric cancer [12]. Therefore, the search for substances capable of gastric ulcer prevention and treatment without causing side effects has intensified. Natural products have gained space in recent decades [13].

Vanillin, or 4-hydroxy-3-methoxybenzaldehyde, is an aldehyde of molecular formula C_8_H_8_O_3_ and molecular weight 152 Da that is commonly used as a flavoring agent [14]. Vanillin, which can be found in pods of the orchid *Vanilla planifolia* as a glycoside, is considered an antimicrobial and antioxidant agent [15]. In addition, its anti-inflammatory [16], antimutagenic [17], and antitumor [18] activities have been described. Al-Asmari et al. [19] described the gastroprotective effect of vanillin, which seems to be due to the inhibition of gastric acid secretion and acidity. We proposed to deepen the knowledge about vanillin gastroprotective activity, aiming to search for an anti-inflammatory activity responsible for the gastroprotective effect.

## 2. Materials and Methods

### 2.1. Animals

We used 35 male Wistars rats, weighing 220–250 g (eight to ten weeks old). The rats were housed in ventilated racks with food and water ad libitum, under controlled temperature, humidity, and lighting. The rats were fasted for ten hours before gastric ulcer induction. We made all efforts to avoid animal suffering. Institutional Ethics Committee in the Use of Animals (CEUA) approved the experimental protocols (permit number 1103, 9 January 2018), which followed the recommendations of the Canadian Council on Animal Care.

### 2.2. Ethanol-Induced Gastric Ulcers

After fasting, the rats were distributed into five groups (n = 7) and orally treated (gavage) with vanillin (25, 50, or 100 mg/kg), vehicle (8% Tween 80 at 10 mL/kg, as the negative control group), or carbenoxolone (100 mg/kg, as the positive control group). After sixty minutes, the rats orally received 5 mL/kg of absolute ethanol. After sixty minutes, the rats were euthanized by anesthetic deepening, using ketamine and xylazine (180 and 25 mg/kg, respectively, by intraperitoneal injection) [20]. Their stomachs were removed, opened along the greater curvature, washed with water, and scanned into two glass plates, which made it possible to measure the ulcer area (mm^2^) using AVSoft BioView software (AVSoft Systems Technology). Afterwards, samples of each stomach were destined for histological analysis or frozen at −80 °C until further analyses were performed.

We used carbenoxolone as the reference drug due to its ability to decrease the severity and number of gastric lesions produced by ethanol ingestion in addition to enhancing the mucus secretion.

The lowest effective dose of vanillin was determined by statistical analyses of the ulcer areas (ANOVA followed by Tukey’s test) and the samples from this group were used for the upcoming analyses.

### 2.3. Histological Analysis

Samples of the stomachs were fixed in 10% phosphate-buffered formalin, processed, embedded in paraffin, and cut into 5 μm sections using a microtome. The histological slides were then stained with hematoxylin and eosin (HE) or periodic acid-Schiff’s reagent (PAS) for histological analysis under a microscope.

### 2.4. Measurement of Cytokine Levels

We processed the stomach samples in phosphate-buffered saline (1:10) in the range of pH 7.2–7.4, centrifuged the stomach homogenate (10,000 rpm, 4 °C, 5 min), and measured the levels of the inflammatory cytokines TNF-α, IL-6, IL-1β, and IFN-γ, and the anti-inflammatory cytokine IL-10 using ELISA kits (R&D Systems).

### 2.5. Gene Expression Analysis by Real-Time, Quantitative PCR (qPCR)

According to the instructions, we used 1 g of stomach tissue for total RNA extraction, using TRI Reagent (Sigma-Aldrich, St. Louis, MO, USA). For the cDNA synthesis, 700 ng of total RNA was used, and the process was performed using iScript gDNA Clear cDNA Synthesis Kit and amplified using iTaq Universal SYBR Green Supermix (Bio-Rad Laboratories, Hercules, CA, USA). The samples were analyzed in duplicate. Ct values were obtained using a 7900HT Real-Time PCR System (Applied Biosystems, MA, USA), and the fold changes of gene expression were calculated by the 2^−ΔΔCt^ method. We designed the primers (NF-κB, TNF-α, and IL-10) based on rat sequences available at Genbank (NCBI, https://www.ncbi.nlm.nih.gov/genbank/, accessed on 4 January 2021). (Table 1). β-actin was the housekeeping gene.

### 2.6. Levels of Nitrite/Nitrate (Total NOx) in the Stomach

We used the Griess reaction followed by a reduction of nitrous species with vanadium chloride III to examine NOx concentrations [21]. In the Griess reaction, the NO level is measured by determining nitrate and nitrite concentrations in the gastric tissue, using the enzyme nitrate reductase to convert nitrate to nitrite. Hence, the total nitrite/nitrate is considered an indirect NO production marker [22]. The samples were centrifuged at 15,000× *g* at 4 °C for 15 min; the supernatant was collected and incubated with 100 μL of a saturated solution of vanadium chloride III for nitrate reduction. After incubation, 50 μL of a 1% sulphanilamide solution in 5% phosphoric acid was added and plate-incubated. Then, 50 μL of a 0.1% N-(1-naphthyl)-ethylenediamine dihydrochloride solution was added. Absorbance was read in a spectrophotometer (535 nm), and NOx concentration was calculated using a standard curve of sodium nitrite. The NOx levels are expressed as μmol/100 mg tissue.

### 2.7. Statistical Analysis

We analyzed the parametric data using a one-way analysis of variance (ANOVA), followed by Tukey’s or Dunnett’s test, and the non-parametric data using Kruskal–Wallis followed by Dunn’s test, using GraphPad Prism software. We present the parametric results as the mean ± standard error of the mean (s.e.m.) and the nonparametric results as the mean. We considered a value of *p* < 0.05 significant.

## 3. Results

### 3.1. Effect of Vanillin on Ethanol-Induced Gastric Ulcer

Gross examination of the stomachs indicated that the oral treatment with vanillin attenuated the number and the length of hemorrhagic bands that characterize the ethanol-induced gastric ulcer, which could be seen in the vehicle group.

**Figure 1:**
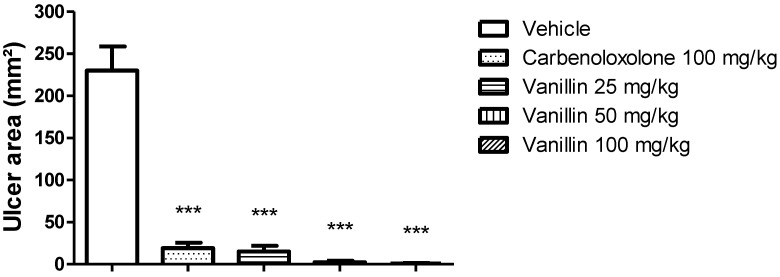
Gastric ulcer area of rats orally treated with vehicle (8% Tween 80), carbenoxolone (100 mg/kg), or vanillin (25, 50, or 100 mg/kg) before ethanol oral administration. ANOVA, Dunnett’s test, *p* < 0.001 in comparison to the vehicle group.

Oral treatment with carbenoxolone and vanillin showed significant (*p* < 0.0001) inhibition of gastric ulcer formation. Carbenoxolone inhibited ulcer formation by 92%, while vanillin inhibited it by 93%, 99%, and 100% at 25, 50, and 100 mg/kg, respectively, compared to the vehicle. The ANOVA followed by Tukey’s test showed no statistical difference between the three doses of vanillin; thereby, the lower dose (25 mg/kg) was used for further analysis. The gastroprotection offered by the three doses of vanillin was not different from the gastroprotection offered by carbenoxolone. Figure 1 represents the gastric ulcer areas.

### 3.2. Histological Analysis

**Figure 2:**
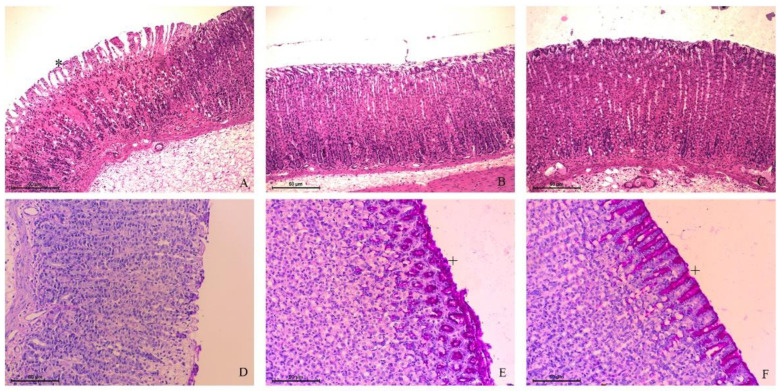
Photomicrography of rat stomachs treated with (**A**,**D**) vehicle (8% Tween 80), (**B**,**E**) carbenoxolone (100 mg/kg), or (**C**,**F**) vanillin (25 mg/kg) before ethanol oral administration. Notice the gastric mucosa is more preserved in (**B**) and (**C**) than in (**A**). * indicates glandular damage (HE staining). In (**D**–**F**), the purple area provides evidence of the mucus layer (+) covering the gastric glands (PAS staining). Bar: 50 µm.

The histopathological examination revealed the typical mucosal damage induced by ethanol [23,24] and the protective activity of vanillin. Ethanol destroyed the simple cylindrical epithelial tissue and disrupted the gastric glands in the vehicle-treated group. There was evident desquamation, thinning, paleness, hemorrhagic damage, intense acidophilia, exfoliation, and erosion of the gastric mucosa. There were inflammatory cells infiltrated in all stomach layers. However, these characteristics were highly attenuated or avoided in rats’ stomachs treated with carbenoxolone or 25 mg/kg vanillin. There were a few points of mucosal desquamation, absence of hemorrhage, and edema in these groups. The treatments were able to preserve the histological architecture of the mucosa, maintaining the structure of gastric pits and lamina propria (Figure 2A–C).

Mucous secretion is the first mucosal defensive factor during gastric injury. We performed PAS staining to visualize the mucopolysaccharides inside the gastric pits. In the vehicle-treated rats, there was a thin mucus layer on the surface of the gastric mucosa, restricted to some points. A purple barrier covered the gastric pits in the carbenoxolone or vanillin-treated rats. This visual result indicated that ethanol administration could not remove the mucus layer after the oral treatments (Figure 2D–F).

### 3.3. Effect of Vanillin in Gastric Inflammation

The quantity of pro-inflammatory cytokines produced by the inflammatory response is directly linked to the development of gastric ulcers. Thus, the downregulation of cytokine production is a desirable target for its treatment [25].

**Figure 3:**
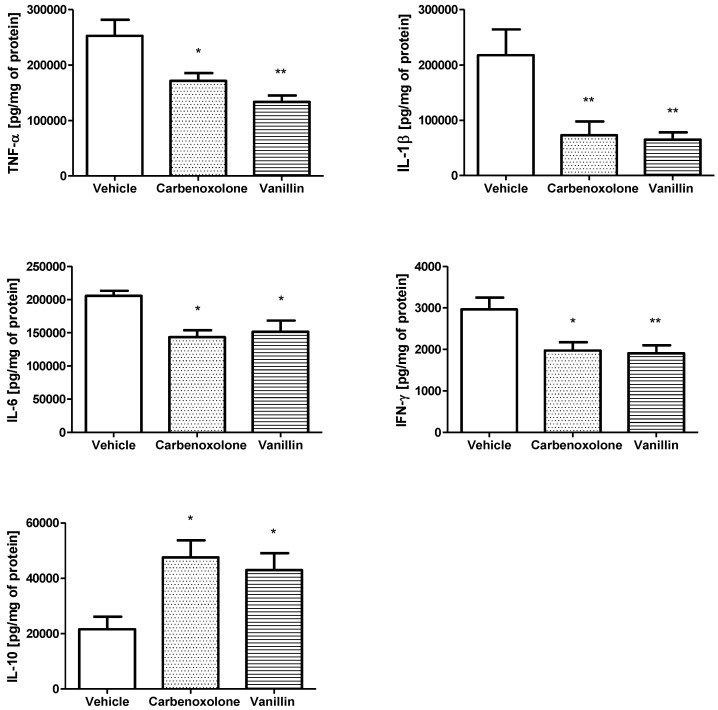
Levels of TNF-α, IL-1β, IL-6, IFN-γ, and IL-10 (pg/mg of protein) in the stomach of rats submitted to ethanol-induced gastric ulcer, after treatment with vehicle (8% Tween 80), carbenoxolone (100 mg/kg), or vanillin (25 mg/kg). ANOVA, Dunnett’s test, * *p* < 0.05, and ** *p* < 0.01 in comparison to the vehicle group.

Vanillin exerted an anti-inflammatory effect in the gastric mucosa by modulating the levels of different cytokines. The oral treatment with vanillin induced a decrease in the level of inflammatory cytokines TNF-α (a reduction of 47%, *p* < 0.001), IL-1β (a reduction of about 70%, *p* < 0.001), IL-6 (a decrease of 26%, *p* < 0.05) and IFN-γ (a decrease of 35%, *p* < 0.001) in comparison to the vehicle group. Furthermore, vanillin nearly doubled the level of the anti-inflammatory cytokine IL-10 (enhanced about 99%, *p* < 0.001). Carbenoxolone treatment (100 mg/kg) was also able to decrease the levels of inflammatory cytokines and increase the level of IL-10 (Figure 3).

### 3.4. Effect of Vanillin in mRNA Expression

**Figure 4:**
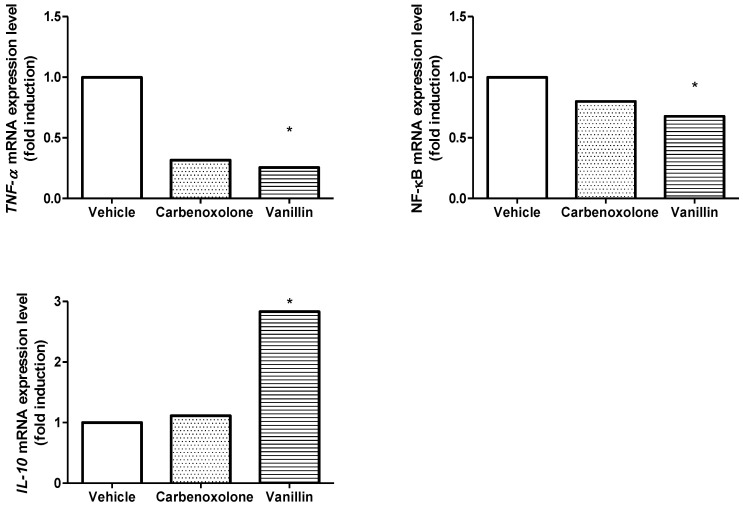
Levels of *Tnfα*, *Nfκb*, and *Il10* mRNA expression in the stomach of rats submitted to ethanol-induced gastric ulcer, after treatment with vehicle (8% Tween 80), carbenoxolone (100 mg/kg), or vanillin (25 mg/kg). Kruskal–Wallis, Dunn test, * *p* < 0.05 in comparison to the vehicle group.

We analyzed the mRNA expression of *Nfκb*, *Tnfα*, and *Il10* genes. Vanillin decreased the level of mRNA expression of *Tnfα* by about 75% and that of *Nfκb* by about 32% (*p* < 0.05 for both). Furthermore, vanillin led to a 2.8-fold increase in the mRNA expression of *Il10* (*p* < 0.05) compared to the vehicle group. Carbenoxolone treatment did not alter the level of mRNA expression of *Nfκb*, *Il10*, or *Tnfα* (Figure 4).

### 3.5. Effect of Vanillin in the Levels of Nitrite/Nitrate (Total NOx)

**Figure 5:**
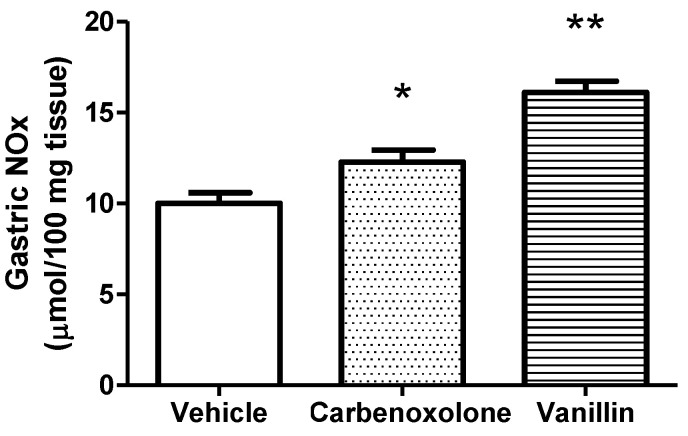
Levels of NOx in the stomach of rats submitted to ethanol-induced gastric ulcer after treatment with vehicle (8% Tween 80), carbenoxolone (100 mg/kg), or vanillin (25 mg/kg). ANOVA, Dunnett’s test, * *p* < 0.05, and ** *p* < 0.01 in comparison to the vehicle group.

The NOx level in the gastric tissue was higher in the carbenoxolone (12.27 ± 0.67 μmol/100 mg tissue, *p* < 0.05) and vanillin-treated groups (16.10 ± 0.62 μmol/100 mg tissue, *p* < 0.01), in comparison to the vehicle group (10.01 ± 0.58 μmol/100 mg tissue) (Figure 5).

## 4. Discussion

In this study, we aimed to investigate the gastroprotective effect of vanillin. We hypothesized that vanillin presents an anti-inflammatory activity that can prevent the development of gastric ulcers induced by absolute ethanol. Ethanol-induced gastric ulcer is the most common experimental ulcer model since it mimics the leading cause of gastric ulcer in humans, ethanol ingestion, aside from screening natural or synthetic products that present an antiulcer effect [26].

We made a dose–effect curve of the vanillin at 25, 50, and 100 mg/kg, which presented similar gastroprotective effects, considering the ulcer area. Therefore, we proceeded with the analyses using the samples of the rats treated with the lower dose.

From a histologic point of view, several studies have described the destructive effects of ethanol administration in the stomach. The mucus barrier covering the gastric epithelial surface is the first line of mucosal defense. The gastric mucus is an adherent gel composed of water and mucin glycoproteins that forms a protective barrier against hydrochloric acid, preventing the proteolytic digestion of the stomach. Furthermore, the mucus entraps bicarbonate ions, maintaining a neutral microenvironment, and prevents the entrance of microorganisms and toxins into the mucosa. The administration of ethanol disrupts the gastric mucus barrier. In contact with the gastric mucosa, ethanol leads to extensive submucosal edema, hemorrhage, desquamation of epithelial cells, and infiltration of inflammatory cells (mainly neutrophils), typical of ethanol-induced gastric ulcer [23]. We could see all these features in the vehicle-treated group. However, vanillin treatment mitigated the damage in histological architecture caused by ethanol, which led us to conclude that the preservation of the mucus barrier covering the gastric pits in vanillin-treated rats is the first defensive factor triggered by vanillin against ethanol challenge. These rats avoided the development of histological lesions.

We macroscopically and microscopically observed the gastroprotective effect of vanillin. In addition to the harmful effects of ethanol in the mucosa and submucosa observed at the microscope, ethanol administration triggers the infiltration of neutrophils. It activates the immune response of T lymphocytes, resulting in a cascade of inflammation, oxidative stress, and apoptosis of the epithelial cells. An intense upregulation of the levels of inflammatory cytokines, such as TNF-α and IL-6, can lead to severe gastrointestinal mucosal damage, augmenting the level of inflammation in the human body [8].

NF-κB is a transcription factor that regulates several physiological roles, such as DNA transcription and cytokine production, and represents one of the main signaling pathways involved in inflammation [27]. The activation of NF-κB involves the phosphorylation of the inhibitor of the κB (IκB) kinases (IKK) complex. The complex phosphorylates the IκB molecules and results in proteasomal degradation of IκB and nuclear translocation of NF-κB, leading to the expression of inflammation-related markers such as TNF-α, IL6, and IL-1β [8]. Al-Asmari et al. [19] demonstrated that vanillin treatment inhibited NF-κB expression in the gastric tissue after ethanol administration using immunohistochemistry reaction. Here, we demonstrated that the gene *Nfκb* was downregulated after oral treatment with vanillin, which reflected a decrease in the production of inflammatory cytokines.

During gastric ulcers, macrophages produce TNF-α and delay the healing in several ways. TNF-α suppresses gastric microcirculation, stimulates neutrophil infiltration, and activates inflammatory signaling pathways, inducing the production of other inflammatory cytokines and NF-κB activation, amplifying its production [26,28].

IL-6 is a pro-inflammatory cytokine that plays a central role in acute inflammation. A high level of IL-6 activates neutrophils, macrophages, and lymphocytes at the site of inflammation, boosting the production of inflammatory mediators and aggravating gastric mucosal injury [29]. The pro-inflammatory cytokine IL-1β also plays a pivotal role in the acute inflammatory response [30].

IFN-γ is a cytokine produced by activated T cells and NK cells. The level of IFN-γ is high in gastric ulcers. IFN-γ acts synergistically with TNF-α, triggering effects on gastric ulcers, including apoptosis, neutrophil infiltration, the release of oxygen free radicals, and other pro-inflammatory cytokines, leading to the destruction of cell membranes and gastric tissue [25].

Consistent with previous studies [31], our data indicated that the oral administration of ethanol triggers a local inflammatory response, increasing the level of inflammatory cytokines in the gastric tissue. However, vanillin reversed this harmful effect, decreasing the inflammatory markers TNF-α, IL-6, IL-1β, and IFN-γ, and augmenting the anti-inflammatory cytokine IL-10. The results of mRNA expression analyses showed that vanillin decreased mRNA expression of *Tnfα* and *Nfkb*, and increased mRNA expression of *Il10*. These data validated the anti-inflammatory effect of vanillin, which is responsible, at least in part, for the vanillin gastroprotective effect.

Several studies have shown that NO levels are involved in maintaining healthy gastric mucosa, although NO can act paradoxically. NO generated from endothelial nitric oxide synthase (eNOS) stimulates vasodilation, scavenges free radicals, decreases the secretion of gastric juice, and relieves the aggregation of leucocytes, increasing mucus production and resulting in the restitution of epithelial tissue integrity. In contrast, NO generated from inducible nitric oxide synthase (iNOS) triggers harmful effects in the tissue through the formation of oxygen free radicals. We found that vanillin oral treatment increased NO levels in the gastric tissue [22,32]. The increased NO levels seem to be a mechanistic effect of vanillin, as several studies have found this result. For example, vanillin normalized NO levels in the psoas muscle and the testis of diabetic rats [33,34] and in the kidneys of rats with cisplatin-induced nephrotoxicity [35].

## 5. Conclusions

Our results provided evidence for the gastroprotective activity of vanillin in rats. This effect is related to an anti-inflammatory effect, mainly inhibiting the NF-κB pathway, resulting in decreased cytokine production. As a promising antiulcer phytomedicine, vanillin could maintain gastric mucosal integrity and support mucus production. Further studies are needed to deepen the comprehension of the vanillin gastroprotective effect.

## Figures and Tables

**Table 1:** Specifications of primers used in qRT-PCR reactions.

Target Gene	Primer sequence 5′–3′	Amplify Length (bp)	Annealing Temperature	NCBI Reference Sequence
*TNFα*	F: ATGGGCTCCCTCTCATCAGT		60 °C	NM_012675.3
	R: TGGTTTGCTACGACGTGGG	100		
*Nfκb*	F: CCTCATCTTTCCCTCAGAGCC		60 °C	NM_199267.2
	R: CGCACTTGTAACGGAAACGC	98		
*Il10*	F: GACGCTGTCATCGATTTCTCC		60 °C	NM_012854.2
	R: GCTCCAAGACAAAGGTGTCTAC	95		
*βactin*	F: CCCTGGCTCCTAGCACCAT		60 °C	NM_031144.3
	R: GATAGAGCCACCAATCCACACA	80		

F: forward sequence; R: reverse sequence; bp: base pairs.

## Data Availability

Data will be available upon request.

## References

[1] Alzokaky A.A., Abdelkader E.M., El-Dessouki A.M., Khaleel S.A., Raslan N.A. (2020). C-phycocyanin protects against ethanol-induced gastric ulcers in rats: Role of HMGB1/NLRP3/NF-κB pathway. Basic Clin. Pharmacol. Toxicol..

[2] Chen H., Liao H., Liu Y., Zheng Y., Wu X., Su Z., Zhang X., Lai Z., Lai X., Lin Z.-X. (2015). Protective effects of pogostone from Pogostemonis herba against ethanol-induced gastric ulcer in rats. Fitoterapia.

[3] Omar H., Nordin N., Hassandarvish P., Hajrezaie M., Azizan A.H.S., Fadaeinasab M., Majid N.A., Abdulla M.A., Hashim N.M., Ali H.M. (2017). Methanol leaf extract of Actinodaphne sesquipedalis (Lauraceae) enhances gastric defense against ethanol-induced ulcer in rats. Drug Des. Devel. Ther..

[4] Albaayit S.F.A., Abba Y., Abdullah R., Abdullah N. (2016). Prophylactic effects of Clausena excavata Burum. f. leaf extract in ethanol-induced gastric ulcers. Drug Des. Devel. Ther..

[5] Friedman G. (2016). Gastroenterology disease and lifestyle medicine. Lifestyle Medicine.

[6] Boligon A.A., de Freitas R.B., de Brum T.F., Waczuk E.P., Klimaczewski C.V., de Ávila D.S., Athayde M.L., de Freitas Bauermann L. (2014). Antiulcerogenic activity of Scutia buxifolia on gastric ulcers induced by ethanol in rats. Acta Pharm. Sin. B.

[7] Chatterjee M., Saluja R., Kanneganti S., Chinta S., Dikshit M. (2007). Biochemical and molecular evaluation of neutrophil NOS in spontaneously hypertensive rats. Cell. Mol. Biol..

[8] Yoo J.-H., Park E.-J., Kim S.H., Lee H.-J. (2020). Gastroprotective Effects of Fermented Lotus Root against Ethanol/HCl-Induced Gastric Mucosal Acute Toxicity in Rats. Nutrients.

[9] Hossen M.J., Hong Y.D., Baek K.-S., Yoo S., Hong Y.H., Kim J.H., Lee J.-O., Kim D., Park J., Cho J.Y. (2017). In vitro antioxidative and anti-inflammatory effects of the compound K-rich fraction BIOGF1K, prepared from Panax ginseng. J. Ginseng Res..

[10] Paulrayer A., Adithan A., Lee J.H., Moon K.H., Kim D.G., Im S.Y., Kang C.-W., Kim N.S., Kim J.-H. (2017). Aronia melanocarpa (black chokeberry) reduces ethanol-induced gastric damage via regulation of HSP-70, NF-κB, and MCP-1 signaling. Int. J. Mol. Sci..

[11] Golbabapour S., Hajrezaie M., Hassandarvish P., Abdul Majid N., Hadi A.H.A., Nordin N., Abdulla M.A. (2013). Acute toxicity and gastroprotective role of M. pruriens in ethanol-induced gastric mucosal injuries in rats. Biomed Res Int..

[12] Cheung K.S., Chan E.W., Wong A.Y.S., Chen L., Wong I.C.K., Leung W.K. (2017). Long-term proton pump inhibitors and risk of gastric cancer development after treatment for Helicobacter pylori: A population-based study. Gut.

[13] Liang J., Dou Y., Wu X., Li H., Wu J., Huang Q., Luo D., Yi T., Liu Y., Su Z. (2018). Prophylactic efficacy of patchoulene epoxide against ethanol-induced gastric ulcer in rats: Influence on oxidative stress, inflammation and apoptosis. Chem. Biol. Interact..

[14] Walton N.J., Mayer M.J., Narbad A. (2003). Vanillin. Phytochemistry.

[15] Bezerra C.F., Camilo C.J., Do Nascimento Silva M.K., de Freitas T.S., Ribeiro-Filho J., Coutinho H.D.M. (2017). Vanillin selectively modulates the action of antibiotics against resistant bacteria. Microb. Pathog..

[16] Lee Y., Kwon J., Khang G., Lee D. (2012). Reduction of inflammatory responses and enhancement of extracellular matrix formation by vanillin-incorporated poly(lactic-co-glycolic acid) scaffolds. Tissue Eng. Part A.

[17] Ho K., Yazan L.S., Ismail N., Ismail M. (2009). Apoptosis and cell cycle arrest of human colorectal cancer cell line HT-29 induced by vanillin. Cancer Epidemiol..

[18] Pedroso L.S., Fávero G.M., de Camargo L.E.A., Mainardes R.M., Khalil N.M. (2013). Effect of the o-methyl catechols apocynin, curcumin and vanillin on the cytotoxicity activity of tamoxifen. J. Enzym. Inhib. Med. Chem..

[19] Al Asmari A., Al Shahrani H., Al Masri N., Al Faraidi A., Elfaki I., Arshaduddin M. (2016). Vanillin abrogates ethanol induced gastric injury in rats via modulation of gastric secretion, oxidative stress and inflammation. Toxicol. Rep..

[20] Robert A., Nezamis J.E., Lancaster C., Hanchar A.J. (1979). Cytoprotection by prostaglandins in rats: Prevention of gastric necrosis produced by alcohol, HCl, NaOH, hypertonic NaCl, and thermal injury. Gastroenterology.

[21] Miranda K.M., Espey M.G., Wink D.A. (2001). A rapid, simple spectrophotometric method for simultaneous detection of nitrate and nitrite. Nitric Oxide.

[22] El-Rady A., Nessren M., Dahpy M.A., Ahmed A., Elgamal D.A., Hadiya S., Ahmed M.A., Sayed Z.E.-A.A., Abdeltawab D., Abdelmohsen A.S. (2021). Interplay of Biochemical, Genetic, and Immunohistochemical Factors in the Etio-Pathogenesis of Gastric Ulcer in Rats: A Comparative Study of the Effect of Pomegranate Loaded Nanoparticles Versus Pomegranate Peel Extract. Front. Physiol..

[23] de Souza M.C., Vieira A.J., Beserra F.P., Pellizzon C.H., Nóbrega R.H., Rozza A.L. (2019). Gastroprotective effect of limonene in rats: Influence on oxidative stress, inflammation and gene expression. Phytomedicine.

[24] Sidahmed H.M.A., Vadivelu J., Loke M.F., Arbab I.A., Abdul B., Sukari M.A., Abdelwahab S.I. (2019). Anti-ulcerogenic activity of dentatin from clausena excavata Burm. f. against ethanol-induced gastric ulcer in rats: Possible role of mucus and anti-oxidant effect. Phytomedicine.

[25] Long X., Zhao X., Wang W., Zhang Y., Wang H., Liu X., Suo H. (2019). Protective effect of silkworm pupa oil on hydrochloric acid/ethanol-induced gastric ulcers. J. Sci. Food Agric..

[26] Mousa A.M., El-Sammad N.M., Hassan S.K., Abd El Nasser A.M., Hashim A.N., Moustafa E.S., Bakry S.M., Elsayed E.A. (2019). Antiulcerogenic effect of Cuphea ignea extract against ethanol-induced gastric ulcer in rats. BMC Complement. Altern. Med..

[27] Fahmy N.M., Al-Sayed E., Michel H.E., El-Shazly M., Singab A.N.B. (2020). Gastroprotective effects of Erythrina speciosa (Fabaceae) leaves cultivated in Egypt against ethanol-induced gastric ulcer in rats. J. Ethnopharmacol..

[28] Luo C., Chen H., Wang Y., Lin G., Li C., Tan L., Su Z., Lai X., Xie J., Zeng H. (2018). Protective effect of coptisine free base on indomethacin-induced gastric ulcers in rats: Characterization of potential molecular mechanisms. Life Sci..

[29] Boutemine I.-M., Amri M., Amir Z.-C., Fitting C., Mecherara-Idjeri S., Layaida K., Sennoun N., Berkane S., Cavaillon J.-M., Touil-Boukoffa C. (2018). Gastro-protective, therapeutic and anti-inflammatory activities of *Pistacia lentiscus* L. fatty oil against ethanol-induced gastric ulcers in rats. J. Ethnopharmacol..

[30] Abdelfattah M.S., Elmallah M.I., Ebrahim H.Y., Almeer R.S., Eltanany R.M., Abdel Moneim A.E. (2019). Prodigiosins from a marine sponge-associated actinomycete attenuate HCl/ethanol-induced gastric lesion via antioxidant and anti-inflammatory mechanisms. PLoS ONE.

[31] Antonisamy P., Subash-Babu P., Alshatwi A.A., Aravinthan A., Ignacimuthu S., Choi K.C., Kim J.-H. (2014). Gastroprotective effect of nymphayol isolated from Nymphaea stellata (Willd.) flowers: Contribution of antioxidant, anti-inflammatory and anti-apoptotic activities. Chem. Biol. Interact..

[32] Wang R., Sun F., Ren C., Zhai L., Xiong R., Yang Y., Yang W., Yi R., Li C., Zhao X. (2021). Hunan insect tea polyphenols provide protection against gastric injury induced by HCl/ethanol through an antioxidant mechanism in mice. Food Funct..

[33] Salau V.F., Erukainure O.L., Olofinsan K.A., Ijomone O.M., Msomi N.Z., Islam M. (2021). Vanillin modulates activities linked to dysmetabolism in psoas muscle of diabetic rats. Sci. Rep..

[34] Salau V.F., Erukainure O.L., Olofinsan K.A., Islam M.S. (2021). Vanillin exerts therapeutic effects against hyperglycemia-altered glucose metabolism and purinergic activities in testicular tissues of diabetic rats. Reprod. Toxicol..

[35] Younis N.N., Elsherbiny N.M., Shaheen M.A., Elseweidy M.M. (2020). Modulation of NADPH oxidase and Nrf2/HO-1 pathway by vanillin in cisplatin-induced nephrotoxicity in rats. J. Pharm. Pharmacol..

